# Non-sensorimotor symptoms in chronic inflammatory demyelinating polyneuropathy

**DOI:** 10.1007/s00415-026-13725-0

**Published:** 2026-03-03

**Authors:** Fabian Klostermann, Oliver L. Steiner

**Affiliations:** 1 Department of Neurology, Motor and Cognition Group, Charité-Universitätsmedizin Berlin, Freie Universität Berlin and Humboldt-Universität zu Berlin, Campus Benjamin Franklin (CBF), Hindenburgdamm 30, 12203 Berlin, Germany; 2 Berlin School of Mind and Brain, Humboldt-Universität zu Berlin, Berlin, Germany; 3 Institute of Psychology, Humboldt-Universität zu Berlin, Berlin, Germany

**Keywords:** Chronic Infl ammatory Demyelinating Polyneuropathy, Non-Sensorimotor Symptoms, Clinical Phenotype

## Abstract

Chronic inflammatory demyelinating polyneuropathy (CIDP) is defined as a dysimmune disorder of the peripheral nervous system (PNS) resulting in sensorimotor deficits. However, an increasing body of data suggests that CIDP also goes along with features, such as autonomic, circadian, fatigue, mood, and subtle cognitive dysfunctions. Some of these non-sensorimotor symptoms (NSMS) challenge the concept of an exclusive PNS disease. Pragmatically, the high prevalence of NSMS calls for a more holistic disease assessment and surveillance to achieve optimal therapeutic results.

## Introduction

Chronic inflammatory demyelinating polyneuropathy (CIDP) is defined as a peripheral nerve disease, characterized by dysimmune attack against the Schwann cell sheath of myelinated axons in somatic nerves [43, 53, 87]. Mostly, patients develop symmetrical, proximal and distal, sensorimotor deficits with a chronic progressive or relapsing course, but purely motor or sensory presentations with distal only or patchy distribution are possible as well [48]. CIDP is widely recognized as a possible cause of neuropathic complaints, deserving mention, because it is considered as an orphan disease—despite considerable uncertainties about its actual prevalence [33, 47]. The disease was firmly conceptualized about 50 years ago [24], but first reports of probable cases of peripheral neuroinflammation date back to the late nineteenth century [87]. Whereas these descriptions did not yet result in the definition of an own disease entity, James Austin came close to this, when in 1958, he reviewed the reports about recurrent polyneuropathies published at that time and compared them with own cases [6]. He already named important CIDP features, such as interstitial nerve edema and proximal or segmental nerve swelling as frequent pathological findings and documented the—for modern readers astonishing—treatment of an own patient with 20 relapses with either placebo or ACTH or cortisone at his discretion, which introduced the knowledge about the steroid sensitivity of some polyneuropathies. In 1975, finally, Dyck and coworkers summarized polyneuropathies with slowed nerve conduction and the clinical as well as histopathological features of CIDP as Chronic Inflammatory Polyradiculoneuritis (CIP) and, in so doing, founded the current concept of a genuine disease without causal relationships to further medical or inherited conditions [24]. In the following decades, the acronym CIDP to denote the primarily demyelinating character of this neuropathy became common [26], and better understanding of the pathophysiology extended the CIDP steroid treatment by different therapies with immunoglobulin *G* formulations, the FcRn antibody efgartigimod, or, in certain situations, plasma exchange [5, 13, 25, 35, 56, 65].

Implicit in the establishment of a medical category and in the quest for adequate treatments is a certain exclusivity in the approach to the condition. In any definition of a disease, the lowest common denominator between affected persons is to be identified to formulate a generalizable description. Naturally, in CIDP, this refers to peripheral nerve dysfunction and its clinical sequelae, mainly numbness and paresis. The same holds true for treatment trials, whose readout parameters are mostly instrumental for the determination of sensorimotor disability, e.g., provided by vigorimetry, or clinical scales, such as INCAT, I-RODS, or MRC sum score [22, 61, 90]. Since CIDP is further diagnosed in a considerable proportion of patients who later turn out to suffer from another condition and many cases of CIDP remain undiagnosed [4, 14, 18], constant efforts are made to align the diagnostic criteria, which are based on the clinical presentation of sensorimotor deficits and neurographic parameters of peripheral demyelination with further supportive aspects from imaging, cerebrospinal fluid, biopsy, or treatment results [34, 86, 88]. In a nutshell, many practical issues back an exclusive focus on the defining lead symptoms of CIDP, particularly with respect to complex treatment decisions. Whether the dose of ongoing therapy needs to be changed, if it can be stopped, or whether a different therapeutic principle should be tried, depends on (next to drug side effects) the evolution of the sensorimotor CIDP symptoms and partly of neurographic parameters [2, 64, 73].

Yet, a too pragmatic approach could narrow the view for some additional disease features, particularly if their expression is subtle. As in other diseases with clear lead symptoms, these tendentially neglected aspects may not consistently prevail in any single patient and if present may by itself not account for much of the entire burden of CIDP. However, since they are probably numerous, they may together unfold a considerable impact on the patients’ quality of life [60, 72]. In this regard, we aim at a critical evaluation of less-recognized sensorimotor and, in particular, non-sensorimotor symptoms (NSMS) in CIDP. Some of these signs are quite anecdotal and a relation to the genuine pathology remains speculative, but others appear so prevalent that their naming as part of the proper disease concept may be considered. In the following, we provide a short overview of these sequelae of CIDP.

### Peripheral manifestations at atypical sites: cranial and truncal affection

Considering that the underlying nerve pathology is less length-dependent in CIDP than in most non-immunological neuropathies and that the disease typically presents with both proximal and distal sensorimotor deficits, symptoms of body parts ‘before the limbs’ are easily conceivable and, indeed, occur. However, as they develop in a minority of cases only, they often raise doubts about the diagnosis. Cranial nerve affection in CIDP is mostly described in single patients or case series [68, 94]. In larger cohorts, they were, however, identified in up to 20% of CIDP patients mostly as facial palsy, but also with bulbar, oculomotor, or trigeminal presentation. Symmetrical deficits were rather related to typical CIDP, whereas unilateral cranial nerve affection prevailed in the multifocal disease variant [78]. Altogether, cranial nerve affection seems less seldom than commonly assumed. This has pragmatic implications, because signs such as tongue fasciculations or atrophy should not only be considered as indicative of other neurological conditions, in particular, motor neuron disease [68]. On a similar note, hypoventilation via phrenic nerve affection and midline body symptoms due to neurogenic dysfunction of the musculus rectus abdominis were seen in CIDP and found responsive to standard CIDP treatments [85, 94].

### Positive symptoms: pain and tremor

Pain and tremor have not always been considered as relevant aspects of CIDP and are still subjects of ongoing research. In contrast to the lead symptoms of CIDP resulting from the loss of sensorimotor nerve functions, they are complex signs due to interactions between central and peripheral processing. As a common denominator of both phenomena, activities in sensorimotor brain networks are shifted toward abnormal steady states, so that one may label them as gain-of-dysfunction symptoms [55, 70, 79].

That the awareness of pain-related problems in CIDP is still rising has different reasons. For example, leading large fiber affection led to the previous assumption that pain was, if at all, of subordinate relevance in this condition [10]. This position appeared supported by a comparative view on other neuropathies, e.g., diabetic or with preponderant small fiber affection, in which pain is often a lead presenting sign [21, 54]. However, systematic assessments showed that around 50% of CIDP patients develop different pain sensations in the course of the disease [15, 31, 63], which in 10–20% are severe and in more than one-third of the cases appear to limit usual everyday life activities [9, 31, 45]. A wrong diagnosis may follow from the belief that pain cannot be the main or primary sign of CIDP, which has been falsified by a number of reported cases [11]. Whereas such presentations are certainly seldom, it follows for usual CIDP care that patients should be explicitly asked for the presence of pain, possibly overshadowed by more overt lead symptoms in clinical routine.

A similar situation can be described with respect to tremor in CIDP, thought to be driven by abnormally synchronized activity in central motor networks emerging as a result of temporally disordered peripheral input [79, 80]. Normally tremor is neither a primary nor a leading CIDP symptom, as has been described for other dysimmune entities, e.g., nodopathies with antibodies against neurofascin-155 [19]. However, on closer examination, tremulous signs were found in the majority of patients with CIDP, more often affecting the upper than the lower extremities [75, 80], and in nearly 20%, this appeared to cause clinically relevant problems [89]. Phenotypically, frequencies of postural and action components in CIDP-related tremor are more variable than in, e.g., essential tremor [79]. This frequency instability, resembling dystonic tremor, has been proposed to reflect a complex interaction of correctional movements for abnormal body postures with oscillatory central signaling [70]. Clinically, it is important to note that tremor often fails to respond to standard CIDP therapies, so that it may require additional drug treatment [79, 89].

### Autonomic neuropathy

In contrast to somatic nerve symptoms affecting more or less unexpected body parts, some occasional deficits go beyond the common description of CIDP as a condition defined by sensorimotor impairments. In particular, autonomic dysfunctions appear to add to the disease burden in some patients. Anecdotal reports and case collections described orthostatic and different bladder voiding or continence problems or further urogenital disturbances, including male as well as female sexual dysfunctions [1, 17, 46, 67, 81]. In larger cohorts, clinically relevant signs of autonomic dysfunction were found in a minority of patients with CIDP despite highly frequent abnormalities identified in technical assessments of autonomic functions. Having said that, single symptoms, e.g., gastrointestinal, genitourinary, or orthostatic problems, had prevalence rates between 15–20% [2, 28, 81], so that the described phenomena might rather be considered as facultative than seldom in CIDP. Since reported incidences strongly diverge even for circumscribed symptoms such as micturitional disturbance, ranging between 2–25% [16, 76], more specific statements remain difficult. This is also underscored by a recent overview of dysautonomic signs in CIDP, based on 12 dedicated studies including 346 CIDP patients [74]. Probably due to incommensurable assessments, reported autonomic abnormalities had a prevalence range from 25% to almost 90% of the patients across these studies. Symptom expression was mostly mild and affected sympathetic as well as parasympathetic functioning, notably without a recognizable correlation to the degree of sensorimotor impairment. Only in three investigations raised data were compared between patients and controls. As group-related abnormalities in CIDP, significant attenuations of the heart rate variability in respiration and standing tests, thermoregulatory sweating [36], the Valsalva ratio and blood pressure responses to sustained hand grip [52], and of orthostatic blood pressure as well as cardiac frequency regulation were found, which in few cases resulted in consciousness loss after prolonged standing [81]. In sum, autonomic dysfunction is a possible aspect of CIDP, which can be severe in a minority of patients [74]. The origin of autonomic disorder in CIDP is not clear, but demyelination of preganglionic sympathetic and parasympathetic axons or entrapment due to nerve swelling may be relevant factors [23, 50]. Besides, it is conceivable that particular symptoms are not driven by the primary CIDP pathology; for example, urogenital dysfunctions may be secondary to CIPD-related problems such as mood change or fatigue reviewed below. However, regardless of mechanisms, a short assessment of autonomic functioning can add relevant information about CIDP patients beyond their sensorimotor condition.

### Non-sensorimotor symptoms in CIDP

Some NSMS of persons with CIDP could be—and probably mostly are—understood as epiphenomena of the disease burden without a relation to the genuine nerval pathology, a notion particularly referring to fatigue, lowered affective state, and sleep problems [12, 45, 59, 71, 72]. Of course, close connections between these symptoms are conceivable. For example, depressiveness due to reduced bodily resources could lead to sleep problems, in turn, facilitating fatigue; or increased efforts to compensate for bodily dysfunctions might provoke fatigue and depressiveness, then disrupting sleep. However, some recent data appear to challenge such straight forward interpretations of at least some of these NSMS.

Probably least surprising in the context of CIDP is the proof of lowered mood, given that most patients notice their symptoms as permanent and, in many cases, progressive. On standard scales, values for depressiveness are often at least doubled in patients compared to controls, which is often of minor or no relevance at an individual level [49, 82, 83]. However, it certainly implies an increased likelihood of relevant depression at any point in time in the course of CIDP [41]. Consequently, it is important to keep the awareness for negative affective developments high.

Fatigue affects 40–80% of persons with CIDP [10, 32, 42, 60, 62, 92]. In so doing, a sensation of lowered physical resources, i.e., motor fatigue, is accompanied by a feeling of reduced mental endurance and resilience [84]. This is worthwhile to note because of findings which suggest that beyond bodily restrictions, the disease is also associated with subtle cognitive change, discussed below.

Poorly recognized CIDP-abnormalities appear to prevail in circadian behaviors. Various studies reported reduced subjective sleep quality and increased daytime sleepiness [27, 72, 84]. Further, actigraphic measurements recently pointed to the existence of sleep dysfunction related to CIDP [82, 83]. These objective data pointed to a fragmentation of sleep with short arousals unrelated to further symptoms, including NSMS such as depressiveness and—as mentioned above—fatigue. Together with the particular clustering of awakenings upon falling asleep and before waking up (middle bedtime phases remaining unimpaired), this appears to indicate that disturbed sleep in CIDP is not a mere epiphenomenon of other CIDP sequelae, but certainly more research is needed to better characterize and understand the nature of sleep disturbances in CIDP. For the time being, one may formulate that CIDP goes along with measurable circadian alterations, but it is not possible to infer on the gravity of these problems from other disease aspects. The relevance of sleep alterations in CIDP is actually not known, but their systematic assessment could help to understand their potential role for the overall disease burden.

Recently, different publications have pointed out subtle cognitive abnormalities in persons with CIDP [49, 77, 82, 83, 93]. These changes are hardly captured by standard screening methods, but become overt in a number of specific tests. So far, their daily life impact remains undetermined, but they merit a word given the mentioned complaints about cognitive fatigue as well as under conceptual aspects. Significantly reduced performances were identified in tasks demanding executive, short and long-term memory, theory of mind, and visuoconstructive functioning [82, 83]. Further, a marked reduction of cognitive processing speed was repeatedly observed [77, 82, 83]. Interestingly, in a recent publication, an association with the level of serum neurofilament light (sNfL) chain was found [30]. As a biomarker of axonal damage, sNfL is elevated in different neurological diseases, among others, in CIDP [29, 40, 51]. This deserves mention under different perspectives. Increased sNfL is not self-evident in CIDP, at least not in early phases of the disease given its primarily demyelinating pathology, and it could be argued that it only occurs if fiber loss becomes a relevant aspect throughout its course. In this scenario, cognitive dysfunction would be rather related to secondary processes than being the consequence of, e.g., neuroinflammation. However, even if this were so, the association between sNfL and cognitive deficits remains enigmatic in the current understanding of CIDP, since peripheral neurodegeneration does not explain an obviously centrally mediated symptom. Accordingly, some central component of the disease may be additionally considered. Worthwhile to mention, sNfL elevation together with a comparable cognitive deficit profile was also described in patients with MS [8, 38], which is interesting, because the spectrum of demyelinating conditions has been suggested to be more fluid than traditionally assumed [3, 20]. This idea has also been formulated in the context of obviously combined central and peripheral demyelinating diseases, labeled as CCPD [39, 58, 66], reported to be associated with particular immunological factors, e.g., antibodies against neurofascin 155 or 186 [7, 91]. Indeed, overrepresentations of central demyelinating lesions were found in CIDP, just as minor peripheral involvement in MS. In this regard, it is further remarkable that in patients with CIDP and a high number of abnormal cognitive test results, cerebrospinal fluid analysis pointed to a leaky blood–brain barrier (BBB), possibly facilitating CNS co-inflammation [93]. However, naturally, these findings and phenotypical analogies cannot prove pathophysiological underpinnings of cognitive change in CIDP and, of course, also factors like psychological distress or circadian disruption may drive the phenomenon. Thus, given only few descriptive data available so far, more research is warranted in this emerging field.

Concerning an impact of CIDP on structures beyond the PNS, retinal change is an additional notable finding. In optical coherence tomography, CIDP patients were shown to have reduced volumes of the retinal ganglion cell layer [37]. Besides this thinning, hypoactivity of so-called intrinsic photosensitive retinal ganglion cells (ipRGCs) was identified [84]. These neurons project to the suprachiasmatic nucleus, which plays a crucial role for the regulation of sleep–wake behaviors, and to further brain areas relevant for cognitive functioning. However, regardless of the question whether ipRGC change is related to the mentioned circadian and cognitive abnormalities, retinal change is interesting per se, since it suggests that CIDP unfolds effects on (at least partially myelinated) CNS neurons via mechanisms still to be determined. As outlined with respect to cognitive change, CIDP and MS-related findings are similar to each other also at retinal levels [57, 69]. In this context, it might be noted on a final remark that vision loss—independent from ipRGC pathology, which does not play a role in image-forming vision—has been described even as presenting feature of CIDP in patients, which showed papilledema and optic neuritis [44].

A summary of the outlined symptoms is provided in Table 1.

**Table 1:** Overview of non-canonical symptoms and findings in CIDP

Category	Prevalence or expression	Evidence	Clinical relevance/contextual awareness
Fatigue	40–80%	High	Established feature; awareness moderate
Pain	~ 50%,	High	Established feature, awareness moderate
Tremor	~ 20%, mostly mild	Moderate	Established feature, awareness low
Autonomic symptoms	25–90%^a^, mostly mild	Moderate	Documented feature, awareness low
Depressiveness	~ doubled compared to controls, mostly mild	Moderate	Possible feature, awareness low
Cognitive abnormality	~ 50%	Emerging	Undetermined impact, awareness low
Sleep dysfunction	frequent (subjective reports)	Emerging	Undetermined impact, awareness low
Retinal change	unknown	Low	Low impact, experimental

^a^Prevalence of autonomic dysfunction varies significantly between technically assessed signs and clinically overt symptoms

## Conclusion and perspective

The clinical picture of CIDP is more complex than its conventional definition as a sensorimotor polyneuropathy suggests. Potential symptoms include, next to sensorimotor neuropathy at cranial or truncal body sites, non-canonical features, including different NSMS. The latter comprise autonomic dysfunction, fatigue, depressiveness, sleep disturbance, as well as subtle cognitive deficits associated with elevated sNfL levels. This appears to challenge the classical concept of CIDP as a disorder strictly confined to the PNS, but for a clearer picture a more systematic and solid clinical database is urgently needed. This includes issues such as potential NSMS associations with specific sensorimotor phenotypes, conceivable modulations by the different CIDP treatment options, and the nature of single NSMS as rather immunologically or psychologically mediated. So far, the data have pragmatical implications, calling for a broader assessment and description of patients beyond sensorimotor dysfunction, to provide individualized therapies to CIDP patients.

## Data Availability

Not applicable.
